# Identification of Dysregulated Competitive Endogenous RNA Networks Driven by Copy Number Variations in Malignant Gliomas

**DOI:** 10.3389/fgene.2019.01055

**Published:** 2019-10-25

**Authors:** Jinyuan Xu, Xiaobo Hou, Lin Pang, Shangqin Sun, Shengyuan He, Yiran Yang, Kun Liu, Linfu Xu, Wenkang Yin, Chaohan Xu, Yun Xiao

**Affiliations:** College of Bioinformatics Science and Technology, Harbin Medical University, Harbin, China

**Keywords:** gliomas, CNV, ceRNA, lncRNA, prognosis

## Abstract

Gliomas represent 80% of malignant brain tumors. Because of the high heterogeneity, the oncogenic mechanisms in gliomas are still unclear. In this study, we developed a new approach to identify dysregulated competitive endogenous RNA (ceRNA) interactions driven by copy number variation (CNV) in both lower-grade glioma (LGG) and glioblastoma multiforme (GBM). By analyzing genome and transcriptome data from The Cancer Genome Atlas (TCGA), we first found out the protein coding genes and long non-coding RNAs (lncRNAs) significantly affected by CNVs and further determined CNV-driven dysregulated ceRNA interactions by a customized pipeline. We obtained 13,776 CNV-driven dysregulated ceRNA pairs (including 3,954 mRNAs and 306 lncRNAs) in LGG and 262 pairs (including 221 mRNAs and 11 lncRNAs) in GBM, respectively. Our results showed that most of the ceRNA interactions were weakened by CNVs in both LGG and GBM, and many CNV-driven genes shared the same ceRNAs in the dysregulated ceRNA networks. Functional analysis indicated that the CNV-driven ceRNA network involved in some important mechanisms of tumorigenesis, such as cell cycle, p53 signaling pathway and TGF-beta signaling pathway. Further investigation of the ceRNA pairs in the communities from the dysregulated ceRNA network revealed more detailed biological functions related to the oncogenesis of malignant gliomas. Moreover, by exploring the association of CNV-driven ceRNAs with prognosis and histological subtype, we found that the copy number status of MTAP, KLHL9, and ELAVL2 related to the overall survival in LGG and showed high correlation with histological subtype. In conclusion, this study provided new insight into the molecular mechanisms and clinical biomarkers in gliomas.

## Introduction

Malignant gliomas are the most common aggressive primary brain tumor (Schwartzbaum et al., 2006; Ostrom et al., 2014). As the most aggressive malignant glioma, glioblastoma multiforme (GBM, WHO grade IV) shows a 5-year survival rate of 5% with the median survival time of 14 months from diagnosis (Parsons et al., 2008). Comparing to GBM, gliomas of WHO (World Health Organization) grade II and III are less aggressive and have been grouped together by The Cancer Genome Atlas (TCGA) as lower grade gliomas (LGGs). Recently, high-throughput studies have proven that copy number variations (CNVs), which are gains or deletions of genomic segments, are considered important risk factors for human cancers (Xi et al., 2011; Park et al., 2017). CNVs are prominent influential factors for gene expression, which may impact the activities of a variety of oncogenic or tumor suppressive pathways (Liang et al., 2016). Many studies have analyzed the impact of CNVs on gene expression phenotypes. For example, Jornsten et al. combined mRNA regulatory relationships with CNV profiles to construct a CNA-driven network using lasso regression and identified driver copy number alterations (CNAs) and explored their effects on transcription in GBM (Jornsten et al., 2011). Park et al. applied a correlation measure to identify significant relationships between copy number variation regions and mRNAs, and characterized the impact of genotypic variations on phenotype in a genome-wide scale (Park et al., 2012). In fact, DNA CNVs not only influenced the expression of protein-coding genes but also affected the expression levels of long non-coding RNAs and miRNAs (Liang et al., 2016).

Recent studies suggested a new layer of miRNA-mediated regulation that RNAs targeted by the common miRNA could “compete” for the miRNAs and thus indirectly regulate each other (Salmena et al., 2011). Such RNAs are called competing endogenous RNAs (ceRNAs), and their miRNA-mediated interactions are referred to as ceRNA interactions. In addition, examples have been already emerging of non-coding RNAs as ceRNAs, such as lincRNA-p21 (Yoon et al., 2012), lincMD1 (Cesana et al., 2011) and linc-RoR (Wang et al., 2013). Experimental evidence has suggested that the aberration of ceRNA interaction can play important roles in tumorigenesis (Tay et al., 2011). Thus, exploring this novel RNA crosstalk will enhance our insight into gene regulatory networks and contribute to a better understanding of human disease (Tay et al., 2014). The existence and strength of ceRNA interactions may vary significantly in different physiological and cellular conditions (e.g., copy number variation). Most ceRNA studies only considered interactions among ceRNAs and miRNAs while overlooking other important gene regulators, such as transcription factors, DNA methylation, and copy number alteration, which would impede our understanding of ceRNA interactions in cancer (Do and Bozdag, 2018). Therefore, incorporating other types of gene expression regulatory factors, namely copy number alteration, to infer condition-specific dysregulated ceRNA interactions in cancer will be meaningful.

Here, we aimed to discover dysregulated ceRNA interactions driven by CNVs in LGG and GBM. We first got the copy number status of each gene and identified over one hundred protein-coding genes and lncRNAs whose expression levels were significantly affected by CNVs in LGG and GBM. Using a customized program, we identified dysregulated ceRNA interactions driven by CNVs and found some interesting features of the dysregulated ceRNA network. Moreover, by systematically characterizing the functions of the CNV-driven ceRNAs, we found their associations with prognosis and histological subtypes.

## Materials and Methods

### Data Source

The DNA copy number (SNP 6.0), mRNA, and miRNA expression data for the LGG and GBM cohorts were collected from the TCGA data portal (https://tcga-data.nci.nih.gov/tcga), and the lncRNA expression data were derived from TANRIC (Li et al., 2015). We extracted 435 LGG and 152 GBM samples with sample-matching copy number data and gene expression data. For DNA copy number data, we determined five types of discretized copy number calls (−2, −1, 0, 1, 2) for genes in LGG and GBM by GISTIC2.0 (Mermel et al., 2011), and genes with no CNV in more than 10% samples were excluded. The gene expression profiles were normalized by log2(tpm+1) and genes with mean expression lower than 30% of samples or with missing values in more than 10% of samples were filtered.

### Identification of CNV-Driven Protein-Coding Genes and lncRNAs

To reduce the influence of noise, we retained high-level amplifications and homozygous deletions discretized by GISTIC2.0 and used the binomial test on the genes that co-existed 2 and −2 status, in which the copy number status with smaller sample size was considered as noise and the copy number status were set to 0 (*P* < 0.05) or deleted (*P* ≥ 0.05). Then, for each protein-coding gene or lncRNA, we divided the gene expression data by copy number status and performed the rank-sum test on the two groups. Genes with concordant changes in copy number status and gene expression were considered to be CNV-driven genes (*P* < 0.05, **Supplementary Table 1**).

### Identification of Dysregulated ceRNA–ceRNA Interactions Driven by CNV

We developed a computational approach to identify dysregulated ceRNA–ceRNA interactions driven by CNVs (**Supplementary Figure 1**). It consisted of the following steps: (1) Obtaining ceRNA–ceRNA interactions in each copy number state. The interactions of mRNA–miRNA and lncRNA–miRNA were obtained from one confidential online miRNA-target databases: StarBase v2.0 (Li et al., 2014). Using the expression profiles of mRNA, lncRNA, and miRNA in each copy number status (i.e. amplification, deletion, and normal), we calculated Pearson correlation coefficient (PCC) between ceRNA pairs as well as mRNA/lncRNA (ceRNA) and miRNA to measure their expression correlations. The ceRNA pairs with significantly positive correlations (adjusted p-value < 0.05) in which each miRNA-ceRNA interaction showed a significantly negative correlation (adjusted p-value < 0.05) were considered as candidate ceRNA triplets in the status. (2) Calculating difference of ceRNA regulation between copy number status. We assumed that the dysregulation caused by CNV will be reflected in the correlations between ceRNA interactions in each candidate ceRNA triplet. So we compared the correlations of ceRNAs in amplification/deletion samples with normal samples to determine the extent of dysregulation. The extent of dysregulation was defined as:

$$∆\text{R}=\left|{\mathrm{cor}}_{v}(\text{ceRNA}1,\text{ceRNA}2)-{\mathrm{cor}}_{n}(\text{ceRNA}1,\;\mathrm{ceRNA}2)\right|$$

where *cor_v_*(ceRNA1, ceRNA2) was the PCC estimated from the amplification/deletion samples and *cor_n_*(ceRNA1, ceRNA2) was from normal samples. If a candidate ceRNA triplet existed only in one copy number status, ΔR was also calculated by using the correlation filtered before. (3) Identifying CNV-driven dysregulated ceRNA–ceRNA interactions. To determine whether ΔR was statistically significant, a permutation test was performed. We randomized the labels of copy number status 1000 times and recalculated the changes of correlation coefficients of each ceRNA pair. A *P* value of 0.05 was used as the cut-off to obtain significantly dysregulated pairs, which were regarded as CNV-driven dysregulated ceRNA–ceRNA interactions. R scripts were available on GitHub (https://github.com/EmeraldG1996/orange-juice/tree/master/ceRNA-interaction).

## Functional Enrichment Analysis

For functional enrichment analysis, we first obtained gene expression profiles of LGG/GBM and matched normal samples from TCGA, and calculated the differential expression of genes. Based on the fold change values, we performed gene set enrichment analysis (GSEA) to discover functions kyoto encyclopedia of genes and genomes (KEGG pathways and GO terms) altered in LGG and GBM, respectively. Then, the hypergeometric test was used to further identify what cancer-related functions the ceRNA network (or community) participated in:

$$p=1-\sum_{k=0}^{m}\frac{{(}_{k}^{M}){(}_{n-k}^{N-M})}{{(}_{n}^{N})}$$

where *N* was the number of genes in the gene expression profiles, *n* was the number of given genes involved in dysregulated ceRNA network or specific community, *M* was the number of genes that participated in cancer-related KEGG pathway/GO term.

### Statistical Analysis of Clinical Data

We downloaded the clinical data of 432 LGG and 124 GBM patients from cBioPortal (http://www.cbioportal.org/). Overall survival curves were constructed by Kaplan–Meier estimation and log-rank tests (*P* < 0.05) were used to identify the significantly survival-related copy number changes. The Cox proportional-hazards regression model was used to investigate the association between the expression of genes and OS. Fisher exact test was performed to detect the clinicopathologic correlates with copy number variations.

## Results

### Identifying DNA Copy Number Variations in LGG and GBM

To systematically evaluate the copy number variations (CNVs) in LGG and GBM, we performed GISTIC2 on TCGA SNP 6.0 array data to get the copy number status of each gene. After filtering segments with copy ratios less than 0.1, 85 putative CNVs in LGG and 65 in GBM were detected, including a total of 152 and 435 patients, respectively. We divided the identified CNVs into two types, i.e., amplification or deletion, for further analysis (**Table 1**, see *Materials and Methods*). Focal amplifications of pathogenic oncogenes were seen in most of the GBM patients. For example, the amplification of PDGFRA was found in 23 patients, and 71 and 28 patients showed EGFR and CDK4 amplification, respectively. We also found some patients harbored focal deletions of tumor suppressor genes, such as CDKN2A (89) and CDKN2B (84). The amplification of oncogenes across LGG was not as extensive as GBM, but focal deletions of CDKN2A/B were also found in LGG, which were considered as negative cell cycle regulators in gliomas (Simon et al., 1999).

**Table 1:** Characterization of genomic CNVs detected in LGG and GBM.

Rank	Genomic Location	Size	No. of Genes	Candidate Gene(s)
**Amplifications (LGG)**				
1	7q32.1	chr7:128,577,665–148,118,090	17	
2	8q24.3	chr8:143,692,404–143,696,833	51	MAF1
**Deletions (LGG)**				
1	9p21.3	chr9:21,329,669–23,898,052	14	CDKN2A,CDKN2B, MTAP,ELAVL2
**Amplifications (GBM)**				
1	4q12	chr4:54,009,788–54,740,715	7	PDGFRA,KIT
2	7p11.2	chr7:53,926,675–57,139,864	17	EGFR,VOPP1
3	12q13.3-14.1	chr12:57,520,417–57,957,269	21	CDK4
**Deletions (GBM)**				
1	9p21.2-21.3	chr9:20,341,664–25,784,562	19	CDKN2A,CDKN2B, MTAP,ELAVL2
2	10q23.31	chr10:87,866,672–87,971,930	1	PTEN

### Different Copy Number Status Affected the Expression of Protein-Coding Genes and lncRNAs

**Figure 1:**
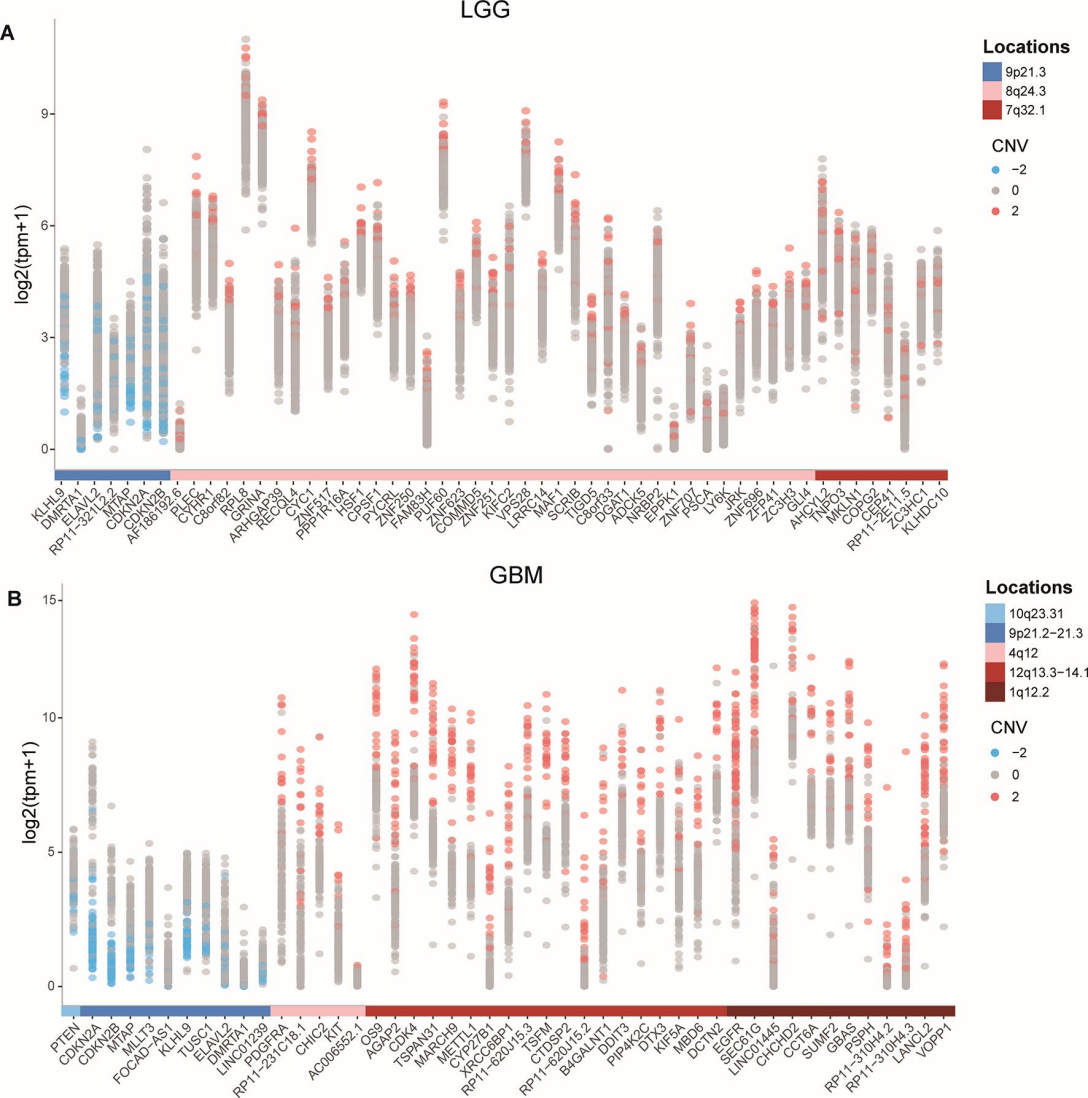
CNVs and the expression of affected protein-coding genes and lncRNAs in GBM and LGG. **(A-B)** Expression of CNV affected genes in GBM **(A)** and in LGG **(B)**.

To identify protein-coding genes and lncRNAs affected by CNVs, we combined copy number data and expression profiles in LGG and GBM. Based on the rank-sum test, we identified genes whose copy number changes (between different copy number statuses) were concordant with changes in their expression (*P* value < 0.05, see Materials and Methods, **Supplementary Table 1**). In LGG, the expression of 52 protein-coding genes and 2 lncRNAs were significantly affected by CNVs, including 46 protein-coding genes and 1 lncRNA showing amplification, and 6 mRNAs and 1 lncRNA associated with deletions. While in GBM, 47 protein-coding genes and lncRNAs were significantly associated with copy number status, including 36 protein-coding genes associated with amplifications, and 9 protein-coding genes and 2 lncRNAs associated with deletions. While our CNV-driven genes were identified between amplification/deletion copy number states and normal state, only several genes were confirmed in previous studies, for example, ELAVL2 in GBM (Bhargava et al., 2017). The genomic localization of these genes showed that the CNVs which significantly affected expression in LGG and GBM could be divided into three and five patterns, respectively (**Figures 1A, B**). In GBM, the CNVs concentrated in 10q23.31 (1), 9p21.2-21.3 (10), 4q12 (5), 12q13.3-14.1 (19), and 7p11.2 (12), consistent with previous reports (Crespo et al., 2012). In these regions, the CNVs of some genes were observed in most patients, including many genes that have been confirmed to be important in the occurrence and development of GBM, such as EGFR, CDKN2A/CDKN2B, and MTAP (Lopez-Gines et al., 2010; Feng et al., 2012; Xu et al., 2017). It has been reported that the deletion of 9p21.3 is related to the occurrence of GBM (Inoue et al., 2004; Alentorn et al., 2015). For LGG, the CNVs concentrated in 9p21.2-21.3 (7), 4q12 (39), 12q13.3-14.1 (8) (**Figure 1A**). Several genes in these regions have been suggested to be important for the prognosis. For example, CDKN2A is an independent predictor of poor survival in diffuse lower-grade gliomas (Aoki et al., 2018).

The expression levels of genes identified as copy number deletion (amplification) were generally decreased (increased) in both LGG and GBM (**Figure 1**), which was consistent with previous reports (Momtaz et al., 2018). At the same time, we found that the degree of expression changes of different genes within one genomic region was not the same. For example, in GBM, the expression of DMRTA1 and LINC01239, which located in the 9p21.3 region, differed by 10 times when copy number changes.

### Identification of the Dysregulated ceRNA Network Driven by CNV

**Figure 2:**
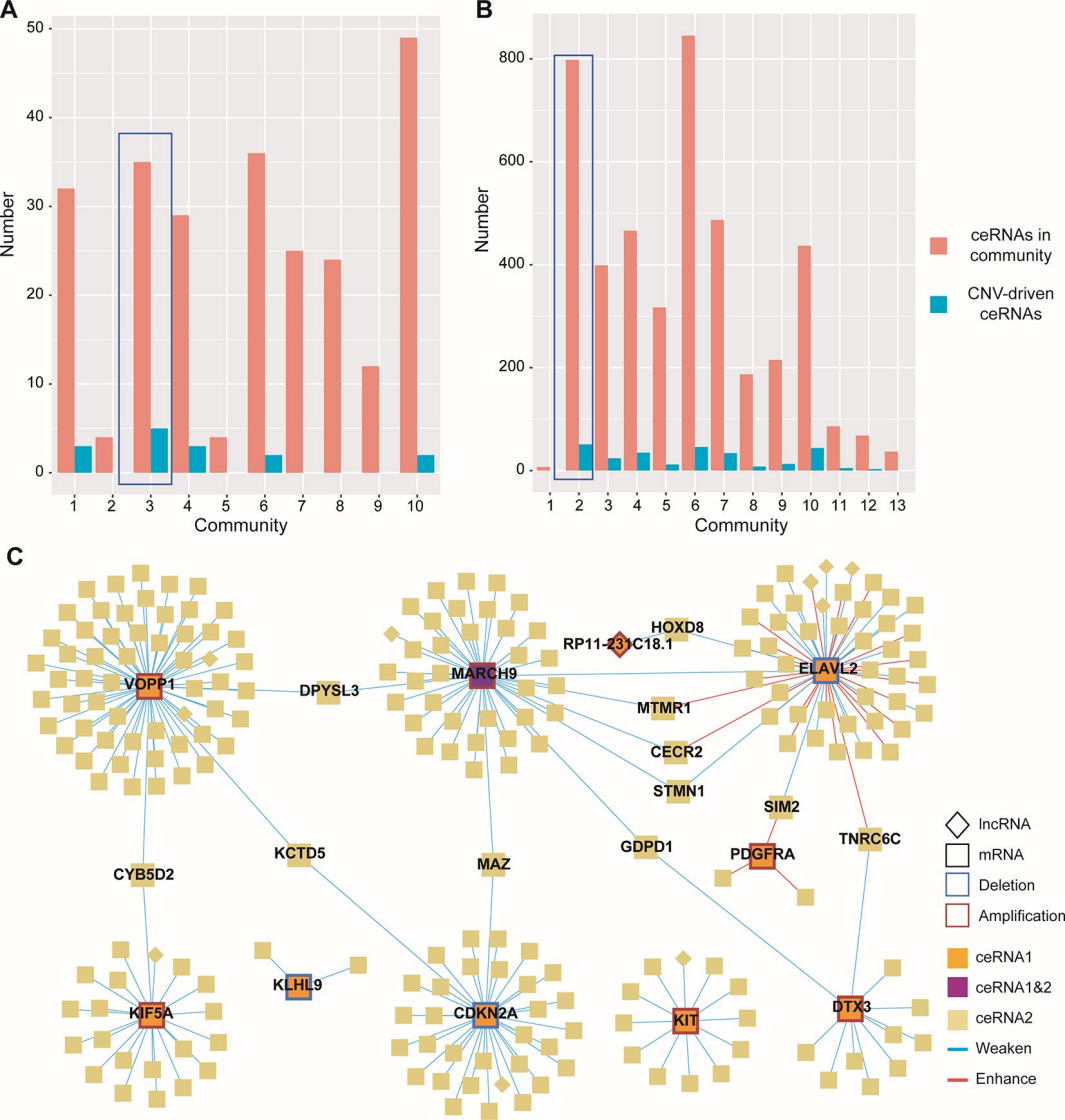
Dysregulated ceRNA pairs driven by CNV. (A–B) Distribution of enhanced and weakened ceRNA pairs in GBM **(A)** and LGG **(B)**. Red for enhanced pairs and blue for weakened pairs. **(C)** Global view of ceRNA network in GBM. The ceRNAs driven by CNV and their ceRNA pairs were colored by orange and light gold, respectively. The CNV-driven ceRNA which was also another CNV-driven ceRNA pair was colored by purple. Square indicated mRNAs and diamond indicated lncRNAs.

Given the lack of exploration of regulatory factors in existing ceRNA studies, we designed a program to identify dysregulated ceRNA interactions driven by CNV (**Supplementary Figure 1**). The program could be roughly divided into three steps. First, the candidate ceRNA triplets were obtained based on the interactions of mRNA/lncRNA-miRNA in LGG and GBM, respectively. Then, to get ceRNA pairs driven by CNV, we calculated the changes of the correlations of ceRNA pairs in each copy number state (amplification/normal or deletion/normal). If CNV increased the correlation, the ceRNA pair was enhanced by CNV. Conversely, the ceRNA pair was weakened by CNV. Last, we used perturbation test to get significant ceRNA pairs driven by CNV (see Materials and Methods, **Supplementary Table 2**). Through the above three steps, we finally obtained 13776 CNV-driven dysregulated ceRNA pairs in LGG, including 3954 mRNAs and 306 lncRNAs. In GBM, we gained 262 copy number-driven dysregulated ceRNA pairs, including 221 mRNAs and 11 lncRNAs (**Figures 2A, B**, **Table 2**).

**Table 2:** The information of dysregulated ceRNA pairs driven by CNV in GBM and LGG.

	CNV-driven ceRNAs		ceRNA pairs	mRNAs	lncRNAs	Enhanced pairs	Weakened pairs
**LGG**	Amplification	34	1488	8031	99	545	943
	Deletion	6	12288	3600	233	549	11739
**GBM**	Amplification	7	102	147	6	29	73
	Deletion	3	160	83	5	3	157

Next, to gain insights into the dysregulated ceRNA interactions caused by CNV, we visualized the ceRNA network with Cytoscape 3.7.0 (Shannon et al., 2003) (**Figure 2C**). By observing the ceRNA network of GBM, we found most of the ceRNA interactions were weakened because of the CNV-driven ceRNAs, and only a few CNV-driven ceRNAs (ELAVL2 and PDGFRA) showed opposite influence (**Figure 2C**). Similar results were also observed in LGG. Interestingly, many CNV-driven genes shared the same ceRNAs in the ceRNA network, and the number of sharing ceRNAs in LGG was larger than GBM. For example, VOPP1 and CDKN2A, which have been proved important in glioma (Xia et al., 2013; Roy et al., 2016), were linked by KCTD5 in GBM (**Figure 2C**). It should be noted that MARCH9, a CNV-driven ceRNA, was also regulated by ELAVL2, and they shared the most ceRNAs (such as MTMR1, STMN1, and CECR2). The interactions between STMN1 and ELAVL2/MARCH9 were weakened by CNV, while in MTMR1 and CECR2 the interactions were weakened by MARCH9 amplification and enhanced by ELAVL2 deletion. In LGG, the ceRNAs shared by MTAP and CDKN2A contained many genes highly associated with gliomas and other cancers, such as IDH1 and CDK4/6 (Cheng et al., 2017). Some studies have shown that co-deletion of CDKN2A and MTAP could be used as markers for glioma stratification, and the deletion of CDKN2A was associated with the expression of CDK4/6 in various tumors (Kaul et al., 2015; Frazao et al., 2018).

### Functional Characterization of Dysregulated ceRNAs Driven by CNV

**Figure 3:**
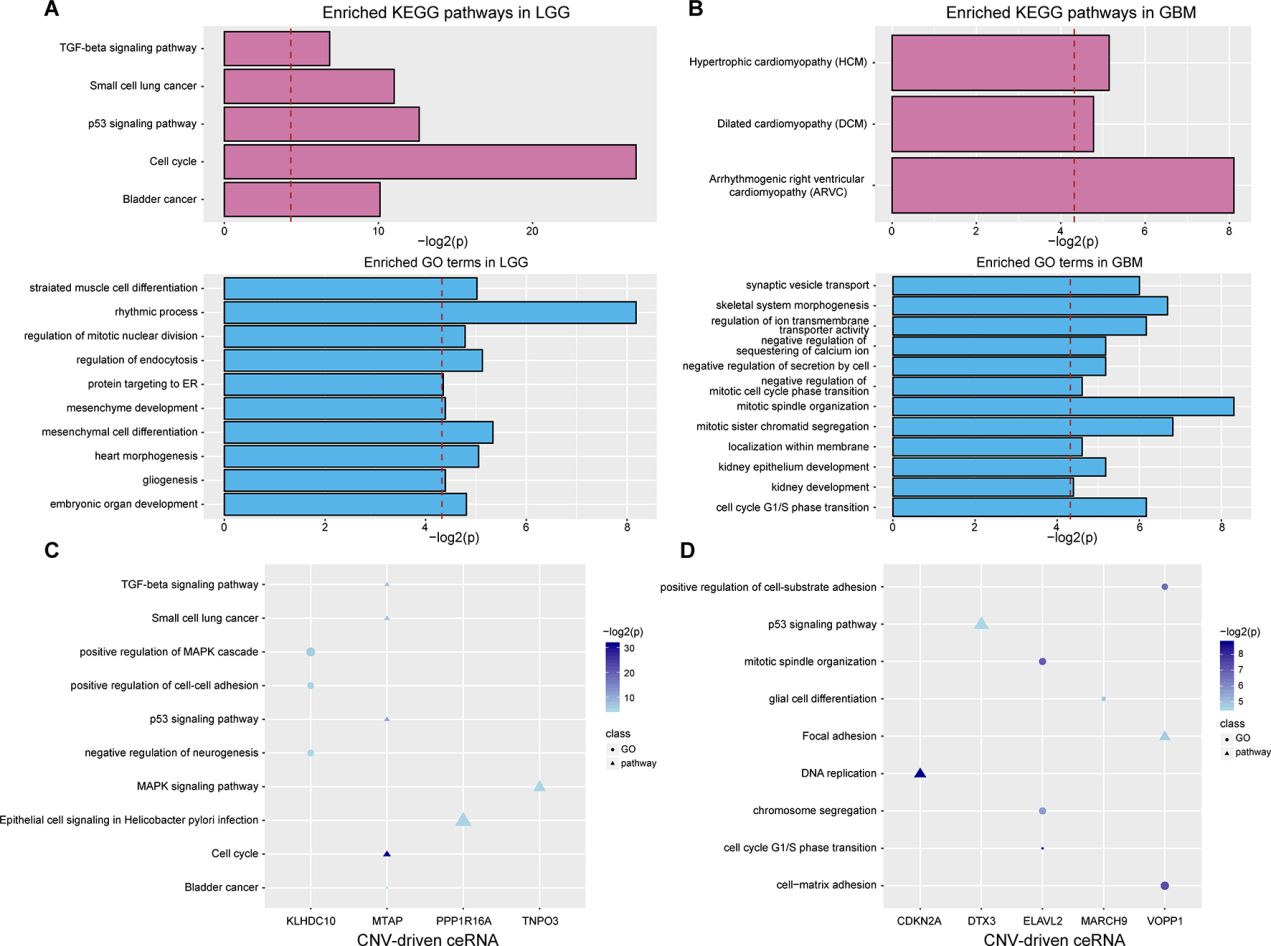
Functional analysis of CNV-driven dysregulated ceRNAs. (A–B) KEGG pathways and GO terms annotated by all dysregulated ceRNAs in LGG **(A)** and GBM **(B)**. The red dotted line indicates that p-value is 0.05. (C-D) KEGG pathways and GO terms annotated by partial CNV-driven ceRNA and its ceRNA pairs in LGG **(C)** and GBM **(D)**. The size of the scatter represents the relative proportion of genes which enriched in the corresponding function.

To evaluate the effects of CNV-driven dysregulated ceRNAs, we used a functional analysis pipeline to characterize their aberrant functions in LGG and GBM, respectively (see Materials and Methods). In LGG, the top significant KEGG pathways, such as cell cycle and p53 signaling pathway, have been shown to play a crucial role in tumor occurrence (**Figure 3A**). For example, the activation of tumor suppressor protein p53 was confirmed to be regulated by CHK-2 kinase in p53 signaling pathway, which indicated that ceRNA network could reflect the mechanism of tumorigenesis (Harris and Levine, 2005). In GBM, dysregulated ceRNAs were primarily enriched in categories related to cell cycle, e.g. cell cycle G1/S phase transition, and cell division, such as mitotic sister chromatid segregation, negative regulation of mitotic cell cycle phase transition and mitotic spindle organization (**Figure 3B**).

We further investigated the functions of ceRNA pairs driven by each CNV with the same approach. By comparing with functions of all dysregulated ceRNAs, we obtained more detailed tumor-related functions in both LGG and GBM. In LGG, an average of three KEGG pathways and four biological processes were identified (*P* < 0.05, **Figure 3C**). The top enriched results not only contained the pathways enriched by dysregulated ceRNAs but also included pathways that regulated cancer development, such as MAPK signaling, which has been shown to significantly promote the proliferation and migration of glioma cells (Wan and Too, 2010; Zhang et al., 2017). Furthermore, we observed ceRNA pairs enriched in the cell cycle, including CDKN2A (a CNV-driven ceRNA), CDK4 and CDK6. It has been proven that cell cycle was mediated by CDKN2A (Aoki et al., 2018), its dysregulation driven by copy number deletion could inhibit CDK4 and CDK6 and thus blocked traversal from G1 to S-phase (Serrano et al., 1993; Kamb et al., 1994). We also found many cancer-related biological functions in GBM, such as p53 signaling pathway, DNA replication as well as GO terms associated with cell cycle (**Figure 3D**). These results demonstrated that more precise regulatory mechanisms related to glioma could be found by annotating dysregulated ceRNAs.

### Exploring Community Structures in the CNV-Driven ceRNA Network

Based on the hypothesis that special topological components in biological networks may provide a new clue to the functional characterization of ceRNAs, we investigated the function of important community structures in the CNV-driven ceRNA network to determine these effects on tumorigenesis (**Figures 2A, B**). Here, modules identified from multi-level optimization of modularity were defined as communities (Song et al., 2017).

**Figure 4:**
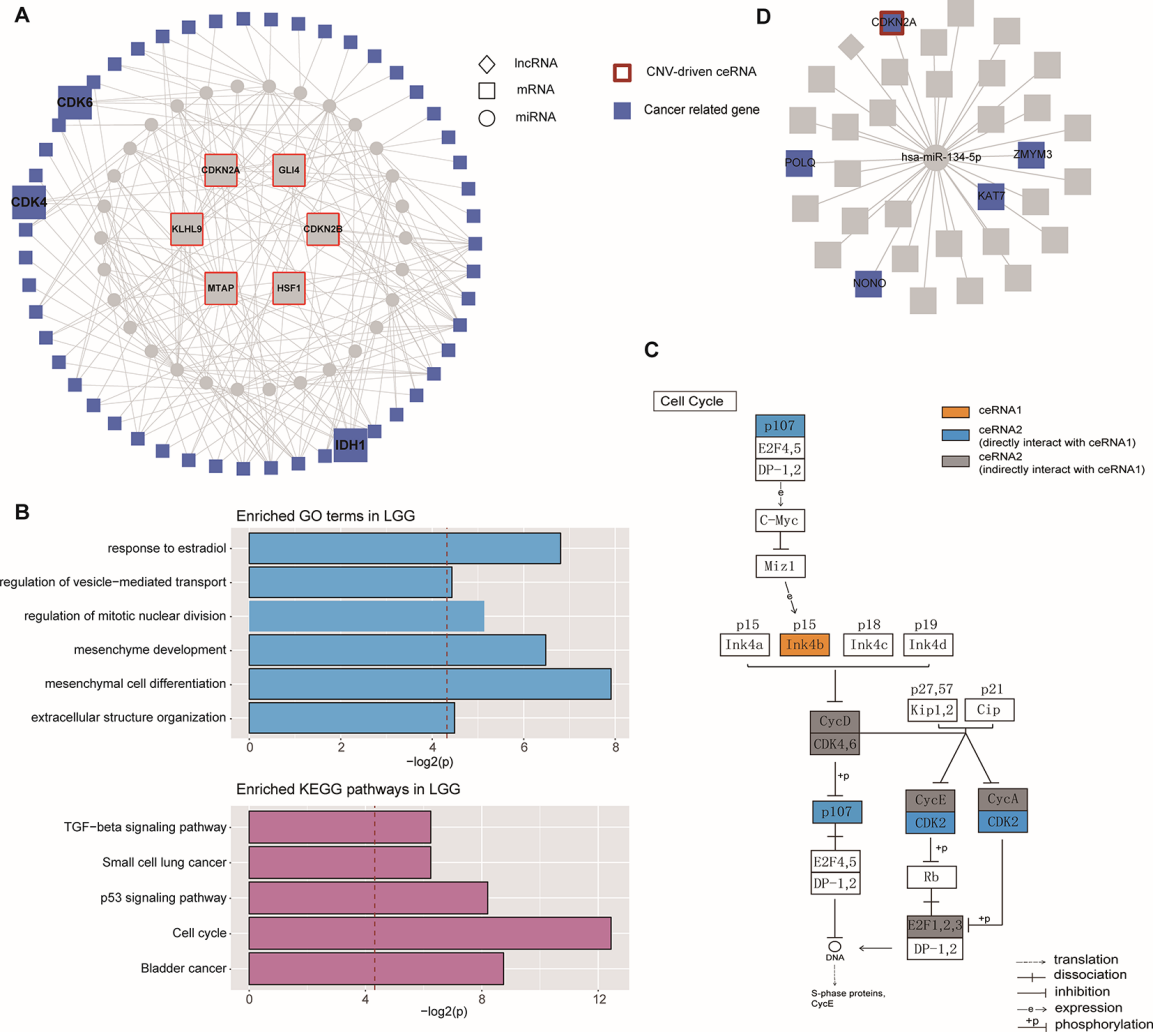
Community analysis in LGG and GBM. **(A)** The architecture of the largest community in LGG. **(B)** Significantly enriched GO terms and KEGG pathways of the largest community in LGG. **(C)** The regulation of ceRNAs involved in cell cycle pathway. **(D)** The architecture of the largest community in GBM.

The largest community in LGG contained 798 nodes, including some glioma-associated genes like IDH1 and CDK4/6 (Cheng et al., 2017), in which most ceRNA pairs were driven by copy number deletion. The functional analysis showed that six GO terms and five KEGG pathways were significantly enriched in this community (p-value < 0.05), such as mesenchyme development, p53 signaling pathway and TGF-beta signaling (**Figure 4**). In this community, BMP-7, as a ceRNA driven by MTAP, has been proved to act as a tumor suppressor that repressed proliferation, self-renewal, and tumor initiation of stem-like glioblastoma cells through suppressing epithelial–mesenchymal transition (EMT) (Zeisberg et al., 2003; Tate et al., 2012). Among all the enriched functions, cell cycle was the most significant (**Figure 4B**), and CDKN2B (Ink4b) drew our attention. As a CNV-driven ceRNA, CDKN2B has been reported to serve as a functional unit in the oncogenesis of malignant gliomas (Shete et al., 2009; Weller et al., 2009), its ceRNA pairs, CDK2 and RBL1, were also annotated in cell cycle and located in the downstream of the pathway (**Figure 4C**). Analogous results were also obtained from the communities of GBM. The largest community of GBM with 34 genes was identified to be relevant to cell cycle-related biological processes (G1/S transition of mitotic cell cycle) and cancer-related pathways (DNA replication) (**Figure 4D**).

### CNV-Driven ceRNAs Associated With Prognosis and Histological Subtypes

**Figure 5:**
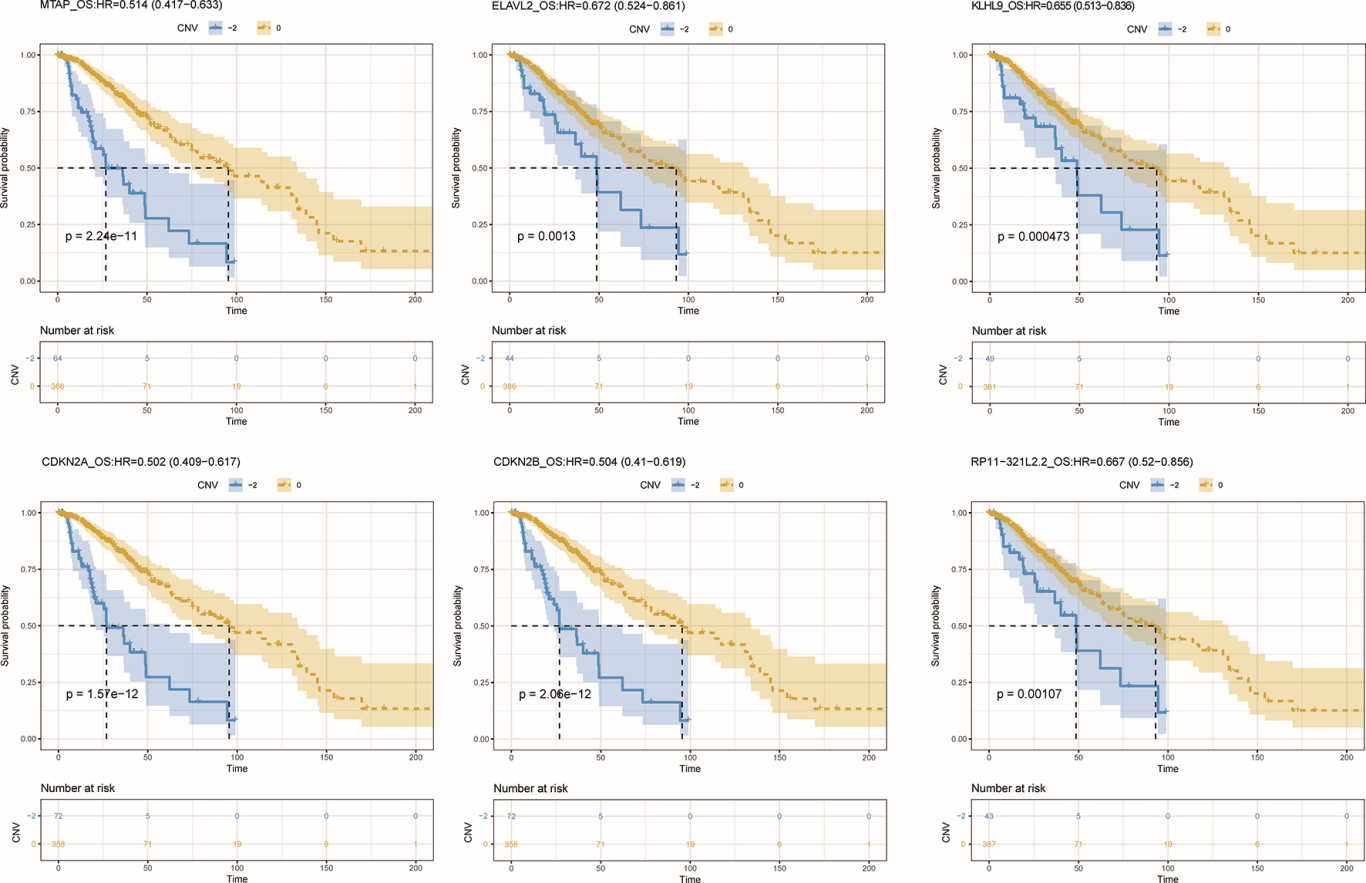
Overall survival among LGG patients (n = 432) stratified by the copy number status of CNV-driven ceRNAs.

To further detect the roles of CNV-driven ceRNAs in prognosis, we assessed whether the effects on the clinical outcome of a CNV-driven ceRNA differed by copy number status. We identified some ceRNAs were significantly related to overall survival in LGG (log-rank test p-value < 0.05, **Figure 5**), but regretfully we did not find any significant results in GBM. For LGG, our results showed that the deletion of MTAP, CDKN2A, and CDKN2B had the worse prognosis (with hazard ratios of 1.946, 1.992 and 1.984, respectively). The dysregulated ceRNA network driven by the deletion of CDKN2B was enriched in Epac1/Rap1 pathway, which was proved to be important in glioma cell death (Moon et al., 2012). By using the Cox proportional hazards regression model, we found that the CNV-driven ceRNAs, such as MTAP, KLHL9, and ELAVL2, whose deletion led to worse overall survival also exhibited significant associations between their expression and survival time (**Table 3**, univariate Cox hazard analysis, *P* < 0.05). Seven of them, for example, KLHL9, showed to be independent prognosis factors (**Table 3**, multivariate Cox hazard analysis, *P* < 0.05).

**Table 3:** Univariate and multivariate Cox hazard analyses in LGG.

CNV-driven ceRNA	Univariate analysis		Multivariate analysis	
	HR (95% CI for HR)	P value	HR (95% CI for HR)	P value
MTAP	0.34 (0.2334–0.4953)	<0.0001	1.0602 (0.4489–2.5036)	0.8939
KLHL9	0.4094 (0.3043–0.5509)	<0.0001	0.3836 (0.207–0.7107)	0.0023
ELAVL2	0.6828 (0.5714–0.8159)	<0.0001	1.3713 (0.9268–2.0289)	0.1142
ZNF517	0.6397 (0.4225–0.9687)	0.0348	0.0913 (0.0286–0.2915)	0.0001
SCRIB	1.5802 (1.1698–2.1345)	0.0029	7.2575 (2.4772–21.2626)	0.0003
PUF60	0.542 (0.3389–0.8668)	0.0106	0.0707 (0.0132–0.3791)	0.002
TNPO3	1.476 (1.0102–2.1566)	0.0442	0.2493 (0.0815–0.7628)	0.0149
VPS28	0.4436 (0.2507–0.785)	0.0052	0.1131 (0.0195–0.6546)	0.015
RP11-2E11.5	0.4738 (0.3411–0.6582)	<0.0001	0.5463 (0.3263–0.9146)	0.0215
GRINA	0.4164 (0.2753–0.6299)	<0.0001	0.4701 (0.1931–1.1442)	0.0963
ARHGAP39	1.4211 (1.0099–1.9998)	0.0438	2.0575 (0.8929–4.7411)	0.0903
CEP41	1.5897 (1.1494–2.1987)	0.0051	1.1195 (0.5523–2.2692)	0.7542
CYHR1	1.5474 (1.1329–2.1134)	0.0061	1.5989 (0.6935–3.6862)	0.2708
KLHDC10	1.592 (1.0146–2.4981)	0.0431	0.9731 (0.2574–3.6795)	0.968
LY6K	0.3638 (0.1954–0.6773)	0.0014	0.7473 (0.292–1.9124)	0.5434
PPP1R16A	1.5943 (1.1601–2.191)	0.004	0.6491 (0.2303–1.8295)	0.4136
ZC3HC1	1.6585 (1.0856–2.5338)	0.0193	1.1166 (0.4487–2.779)	0.8126
ZNF707	1.7057 (1.1677–2.4915)	0.0057	1.6517 (0.6609–4.1278)	0.2829
RECQL4	1.6168 (1.2803–2.0417)	0.0001	0.9333 (0.5146–1.6927)	0.8202

Furthermore, we found that the CNV-driven prognosis factors also showed high correlation with histological subtype (**Table 4**, Fisher exact test, *P* < 0.05). Interestingly, all of the subtype related CNV-driven ceRNAs were located in the deletion region at 9p21. It has been shown that the deletion of 9p21, especially co-deletions of CDKN2A/B and MTAP, could be a marker for different grades of glioma (Frazao et al., 2018). Interestingly, CDKN2B, CDKN2A, MTAP, and KLHL9 also belonged to the largest community in the dysregulated ceRNA network, suggesting their possible role to inhibit the development of glioma together. Besides, we also found a lncRNA, RP11–321l2.2, whose ceRNA pairs were involved in MAPK and PI3K pathways.

**Table 4:** Fisher exact test of histological subtypes and copy number status of CNV-driven ceRNAs.

ceRNA	CNV	Histological subtypes			p-value
		Astrocytoma	Oligoastrocytoma	Oligodendroglioma	
MTAP	0	117	101	150	5.61E-05
	−2	39	12	13	
ELAVL2	0	128	105	155	0.000373
	−2	28	8	8	
KLHL9	0	125	104	154	0.00018
	−2	31	9	9	
CDKN2A	0	111	101	148	3.50E-06
	−2	45	12	15	
CDKN2B	0	110	101	149	8.65E-07
	−2	46	12	14	
RP11-321L2.2	0	129	105	155	0.000736
	−2	27	8	8	

## Discussion

In this study, we provided a comprehensive catalog of dysregulated ceRNA interactions driven by CNV in both LGG and GBM. We identified the expression of protein-coding genes and lncRNAs affected by CNVs and figured out consistent changes of genes in both cancer subtypes. Based on the CNV-driven genes and ceRNA triplets, dysregulated ceRNA networks driven by copy number amplification/deletion were identified in LGG and GBM. We found that CNV could attenuate the interactions between most ceRNA pairs, and the dysregulated ceRNAs driven by CNV were involved in some critical biological functions in glioma. Furthermore, some CNV-driven ceRNAs showed a significant correlation to overall survival, indicating that they may be potential clinical biomarkers of prognosis.

We not only demonstrated that the dysregulated ceRNA network could be influenced by CNV in both LGG and GBM but also obtained some critical biological functions related to the CNV-driven dysregulated ceRNAs. These ceRNAs were significantly enriched in the programs of tumorigenesis, such as cell cycle, p53 signaling pathway. By further functional analysis of each CNV-driven ceRNA sub-network, we identified more detailed tumor-related functions, for example, cell cycle G1/S phase transition. Our study demonstrated a novel finding that the CDKN2B (p15, driven by copy number amplification) could regulate TGF-β signaling pathway in LGG. TGFβR1, which was a ceRNA pair of CDKN2B, is activated by binding with TGF-β (Massague, 1992). Another ceRNA GDNF, a member of TGF-β super-family, has been revealed to strongly induce glioma cell proliferation and migration (Song and Moon, 2006; Ng et al., 2009). These findings could potentially account for the mechanism that TGF-β receptors may be mediated by CDKN2B to influence the glioma occurrence and development. Meanwhile, higher levels of RhoA, another ceRNA member of CDKN2B and a downstream factor in TGF-β/MAPK signaling pathway, can significantly promote glioma cell proliferation and migration (Wan and Too, 2010; Shabtay-Orbach et al., 2015). These results suggest that although the regulation of CDKN2B through TGF superfamily members is not clear, it is worth to determine in the future.

By performing a functional analysis of the largest community in CNV-driven ceRNA network, we could identify key biological functions relevant to LGG pathogenesis. Epithelial–mesenchymal transition (EMT) is known as a facilitator of cellular dissociation and migration, which plays a critical role in cancer metastasis (Cheng et al., 2012; Iwadate, 2016). Our results elucidated a key EMT-related molecule: BMP-7 and discovered a critical ceRNA interaction between MTAP and BMP-7. The ceRNA interactions explain the role of EMT in malignant glioma, which may provide new insight into the mechanism of tumorigenesis. Additionally, the loss of CDKN2B could cause the dysregulation of its relevant community structures, by affecting the expression of its ceRNA partners, including CDK2 and RBL1, and ultimately resulted in cell-cycle dysregulation. These ceRNAs founded by exploring specific community structures could provide new potential therapeutic targets for malignant gliomas.

**Figure 6:**
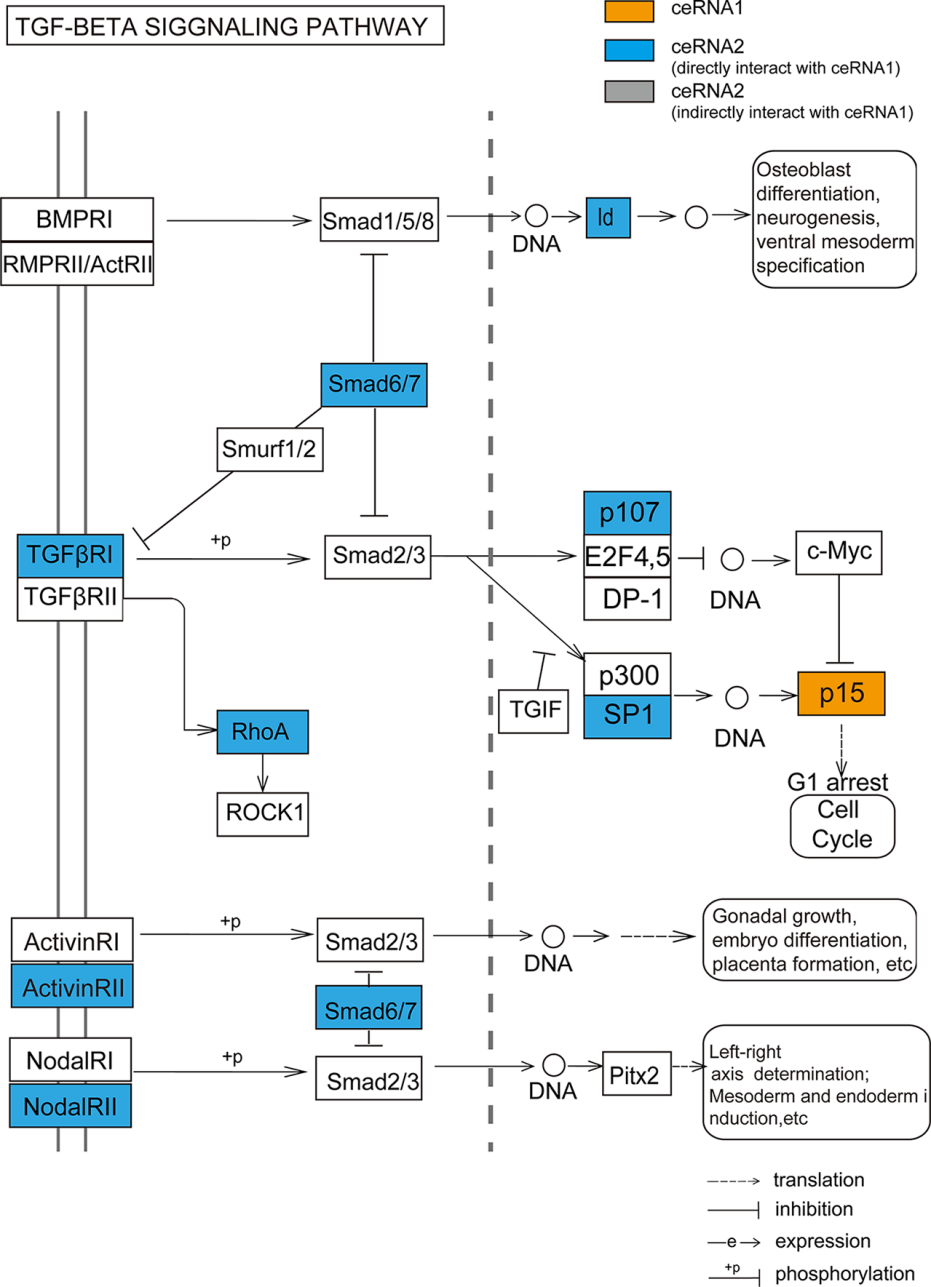
The TGF-beta signaling pathway annotated by ceRNA pairs of CDKN2B (p15). Orange node represents CNV-driven ceRNA. Blue nodes represent the ceRNA members of the CNV-driven ceRNA.

Our study further revealed the putative influence of CNV-driven ceRNAs in clinicopathologic characteristics. By performing a systematic analysis of the CNV-driven ceRNAs with clinical features, we found that the CNVs of some genes (such as MTAP/CDKN2A/CDKN2B/KLHL9) had significant impacts on histological diagnosis and survival in glioma. Functional analysis of CDKN2B through its influenced ceRNA network further revealed that the dysregulation of specific ceRNA networks driven by CNVs could act as prognostic markers of glioma (**Figure 6**). We proposed that the CNV-driven ceRNAs detected to be associated with clinical features may possess clinical functions through regulating other genes by ceRNA networks. The CNV-driven ceRNA network could be used to presume potential prognostic markers of glioma.

## Data Availability Statement

Publicly available datasets were analyzed in this study. This data can be found here: https://tcga-data.nci.nih.gov/tcga.

## Author Contributions

YX, CX, and JX conceived and designed this study. XH, LP, SS, and SH collected and analyzed the data. JX, XH, SS, and SH carried out the method and performed the analysis. YY, KL, and LX helped to analyze the results. JX, XH, SS, and YY participated in the discussion of the project. JX, LP and WY revised the manuscript. All authors reviewed, edited, and

## Conflict of Interest

The authors declare that the research was conducted in the absence of any commercial or financial relationships that could be construed as a potential conflict of interest.

## Supplementary Material

Supplementary Figure 1 The computational approach to identify dysregulated ceRNA–ceRNA interactions driven by CNVs.

Supplementary Table S1 List of the CNV-driven genes.

Supplementary Table S2 List of the significant ceRNA pairs driven by CNV.
