# Supplementary figures and images for: Identification of Dysregulated Competitive Endogenous RNA Networks Driven by Copy Number Variations in Malignant Gliomas
Source: Front Genet. 2019 Oct 25;10:1055. doi: 10.3389/fgene.2019.01055 (PMC6827427; doi:10.3389/fgene.2019.01055)

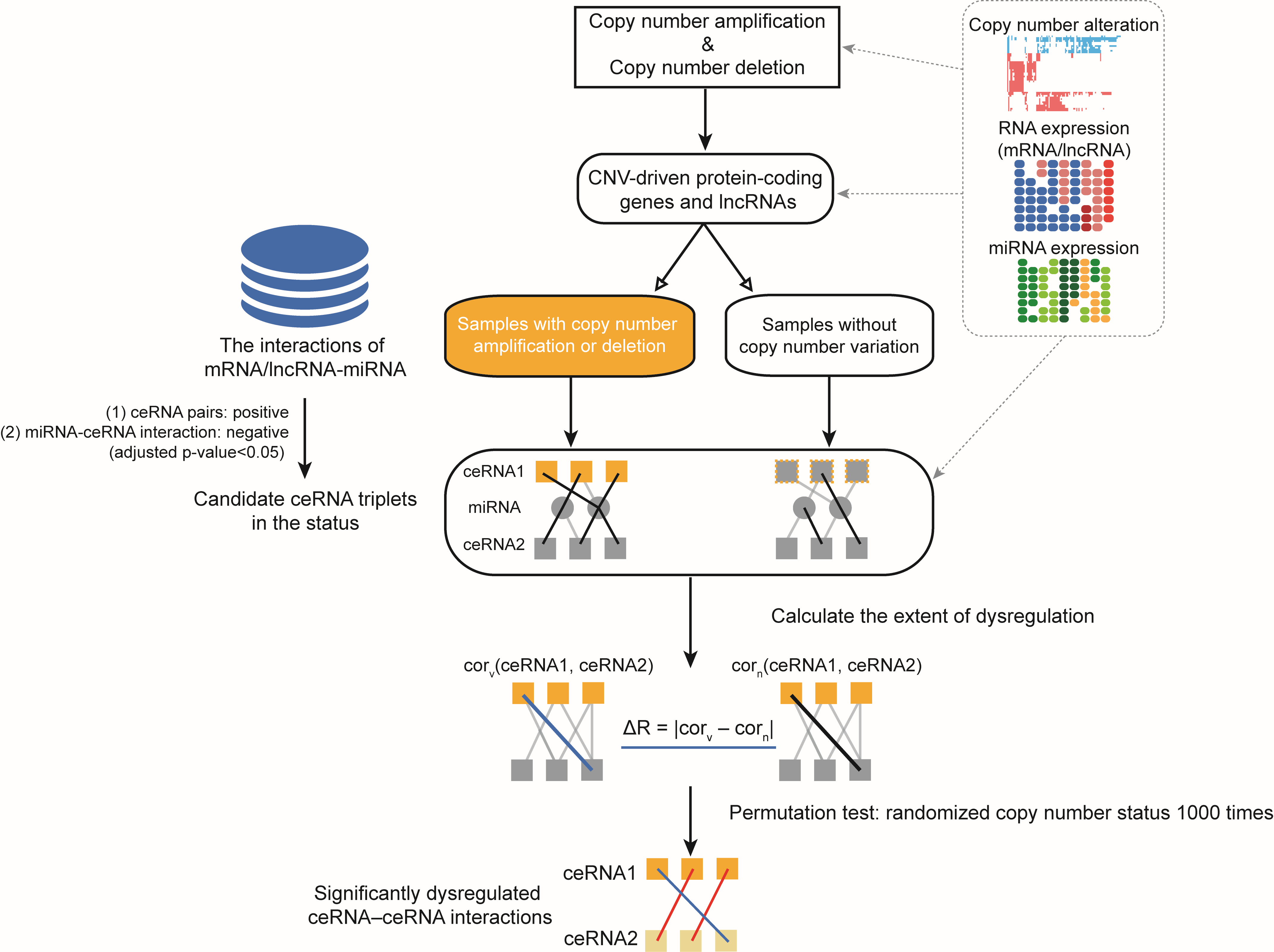

Supplement: Supplementary Figure 1 — The computational approach to identify dysregulated ceRNA–ceRNA interactions driven by CNVs. [file Image_1.tiff]
